# Using spontaneous eye blink-related brain activity to investigate cognitive load during mobile map-assisted navigation

**DOI:** 10.3389/fnins.2023.1024583

**Published:** 2023-02-14

**Authors:** Bingjie Cheng, Enru Lin, Anna Wunderlich, Klaus Gramann, Sara I. Fabrikant

**Affiliations:** ^1^Department of Geography and Digital Society Initiative, University of Zurich, Zurich, Switzerland; ^2^Department of Biopsychology and Neuroergonomics, Technical University of Berlin, Berlin, Germany

**Keywords:** blink-related potentials, cognitive load, mobile map design, assisted navigation, spatial learning

## Abstract

The continuous assessment of pedestrians’ cognitive load during a naturalistic mobile map-assisted navigation task is challenging because of limited experimental control over stimulus presentation, human-map-interactions, and other participant responses. To overcome this challenge, the present study takes advantage of navigators’ spontaneous eye blinks during navigation to serve as event markers in continuously recorded electroencephalography (EEG) data to assess cognitive load in a mobile map-assisted navigation task. We examined if and how displaying different numbers of landmarks (3 vs. 5 vs. 7) on mobile maps along a given route would influence navigators’ cognitive load during navigation in virtual urban environments. Cognitive load was assessed by the peak amplitudes of the blink-related fronto-central N2 and parieto-occipital P3. Our results show increased parieto-occipital P3 amplitude indicating higher cognitive load in the 7-landmark condition, compared to showing 3 or 5 landmarks. Our prior research already demonstrated that participants acquire more spatial knowledge in the 5- and 7-landmark conditions compared to the 3-landmark condition. Together with the current study, we find that showing 5 landmarks, compared to 3 or 7 landmarks, improved spatial learning without overtaxing cognitive load during navigation in different urban environments. Our findings also indicate a possible cognitive load spillover effect during map-assisted wayfinding whereby cognitive load during map viewing might have affected cognitive load during goal-directed locomotion in the environment or vice versa. Our research demonstrates that users’ cognitive load and spatial learning should be considered together when designing the display of future navigation aids and that navigators’ eye blinks can serve as useful event makers to parse continuous human brain dynamics reflecting cognitive load in naturalistic settings.

## 1. Introduction

### 1.1. GPS-based navigation systems and spatial learning

“I move, therefore I am” (Murakami, 2011). We move in space to work, to shop, to travel, and more. Coordinated and goal-oriented movement through an environment is defined as “navigation” (Montello, 2005). Navigation is a fundamental human activity in daily life and used to be an essential skill for our human ancestors for survival. Navigation, especially in novel environments, is a cognitively challenging task that involves numerous cognitive processes, including perception, memorization, and reasoning of places and orientation in space (Montello, 2005). These cognitive processes are supported by multiple brain regions, such as the occipital cortex, the hippocampus, the retrosplenial cortex, and the entorhinal cortex (Ekstrom et al., 2014; Do et al., 2021), and are important for not only navigation but also healthy aging (Coughlan et al., 2018) and spatial reasoning in education (Uttal and Cohen, 2012).

In a digital era, cognitive tasks during navigation are increasingly taken over by GPS-enabled mobile map displays as interfaces to navigation systems that provide automatic self-localization, route planning, and turn-by-turn instructions in real-time (Wenig et al., 2017). Assisted with such mobile map displays at their fingertips, navigators likely follow the route shown on their mobile screens passively, which may limit their active exploration in the environment (Clemenson et al., 2021). With their gazes fixated on the mobile map, navigators tend to allocate less of their attention to the traversed environment (Gardony et al., 2013, 2015; Brügger et al., 2019) and are less likely to actively encode the navigation-relevant environmental information (e.g., landmarks and routes) seen in the traversed environment into their memory (Parush et al., 2007; Clemenson et al., 2021; Sugimoto M. et al., 2022). As a consequence, increased use of GPS-enabled mobile navigation devices has been shown to negatively affect navigators’ spatial learning and their innate spatial skills, as a large body of literature has demonstrated (e.g., Ishikawa, 2019, Ruginski et al., 2019, Dahmani and Bohbot, 2020). Therefore, there is a need to develop GPS-enabled navigation systems that alleviate these negative consequences on a population that is increasingly reliant on mobile maps.

### 1.2. Cartographic design of mobile map displays

Could the self-localization and route-planning options of GPS-enabled navigation assistance be responsible for the abovementioned negative effects or is it due to mobile map design? Cartographers and psychologists approached the problem of spatial deskilling from an interdisciplinary perspective by asking how GPS-enabled mobile map design influences navigators’ wayfinding behavior and spatial learning (Münzer et al., 2012, 2020; Liao et al., 2017; Brügger et al., 2019; Stevens and Carlson, 2019; Keil et al., 2020; Kapaj et al., 2021). Münzer et al. (2012, 2020) examined different mobile map design choices, such as whether a mobile map should dynamically align with the body orientation of the navigator during wayfinding compared to the static north-up orientation of traditional paper maps, and if the viewing perspective of a mobile map should switch between a first-person view (i.e., egocentric perspective) as encountered during navigation or remain in the commonly used birds-eye view of traditional paper maps (i.e., allocentric perspective). The authors concluded that the acquisition of different types of spatial knowledge, such as egocentric and allocentric spatial knowledge, can be facilitated by appropriate map visualization without impeding wayfinding efficiency. Liao et al. (2017) investigated the level of spatial detail or fidelity of depiction with the environment visualized on mobile maps by comparing abstract 2D cartographic maps with realistic-looking 3D satellite image maps. The authors found that while depicted landmarks on both types of mobile map displays supported navigators’ route direction memory at complex intersections, satellite image maps impeded spatial memory building due to visual information overload.

Cartographers have conducted decades of research to provide map design solutions to efficiently and effectively communicate spatial information to support human mobility (for an overview, see Montello, 2002; Ricker and Roth, 2018). The emphasis of this body of cartographic research is on map display design and user interface and user experience (Ricker and Roth, 2018), and how traditional cartographic design principles transfer to the interactive and dynamically updating small mobile map screen (Muehlenhaus, 2013). Until very recently, the field did not directly consider the background and training of the navigator, or the effects on spatial learning (Thrash et al., 2019; Li, 2020). More recently, attention has also been drawn to reduce the adverse effects of GPS-enabled navigation aids on spatial learning, e.g., by geographic information scientists (GIScientists) (Wenig et al., 2017), cognitive scientists (Ruginski et al., 2019), and map user interface (UI/UX) designers (Ricker and Roth, 2018; Thrash et al., 2019; Li, 2020; Fabrikant, 2022). Among the ideas proposed, the appropriate inclusion and display of landmarks on GPS-enabled mobile maps has gained particular traction among cartographers and navigation researchers in GIScience (Raubal and Winter, 2002; Duckham et al., 2010; Credé et al., 2020; Keil et al., 2020; Liu et al., 2022).

### 1.3. Landmark-based navigation and cognitive load

Geographers refer to landmarks as distinctive geographic features in an environment (Richter and Winter, 2014). Landmarks are commonly used as cognitive anchors in space and to structure mental representations of space (Richter and Winter, 2014). Landmarks help navigators to determine their current position and heading (Michel and Ariane, 2020; Yesiltepe et al., 2021), to remember decision points along a path, directions taken on route intersections (Philbeck and O’Leary, 2005; Ligonnière et al., 2021), and to navigate to destinations by retrieving long-term spatial knowledge of landmark relations across traversed environments (Chrastil and Warren, 2013; Epstein and Vass, 2014). Despite the importance of landmarks for navigation and wayfinding, existing mobile map interfaces on navigation systems still provide turn-by-turn instructions that typically refer to metric distance information (e.g., turn left in 200 m).

Although adding landmarks to mobile maps can ease navigation and spatial learning, the visuo-spatial processing of shown landmarks can also require additional cognitive resources and/or distract from the wayfinding task, thereby increasing cognitive load. Cognitive load refers to the total amount of cognitive resources that are used at any given moment for cognitive processing (Sweller, 1988; Baddeley, 2003). With limited cognitive resources, learning performance reaches a plateau (or even drops) when the number of items to be learned exceeds individuals’ limited cognitive capacity. Cognitive load increases as the number of items to be remembered approaches individuals’ cognitive capacity (Sweller, 1988).

The cognitive capacity literature has suggested that humans’ ability of remembering simple visual items with one level of features (e.g., color, shape, orientation, etc.) to be around four items (or chunks; Luck and Vogel, 1997; Vogel et al., 2001; Baddeley, 2003). However, cognitive capacity is not a fixed number, especially when it relates to meaningful and complex real-world objects with multiple visual and spatial features. Recent studies have found that learners tend to remember a higher number of real-world objects (e.g., vases embroidered with visual details on their surface), compared to simple visual items, such as oriented lines and different sized stimuli (for a review, see Endress and Potter, 2014; Brady et al., 2016, 2019; Sahar et al., 2020). This is because learners can integrate features of one object (i.e., ensemble processing) and ascribe meanings to them, and not just memorize individual abstract items (Brady et al., 2019). In an urban navigation context, for example, landmarks such as visually salient buildings in a city typically contain multiple visual (e.g., color, texture, etc.), spatial (e.g., size, shape, orientation at turning points, etc.), and semantic features (e.g., the post office, the school, my home, etc.). Individuals should therefore be able to encode and remember more than 4 chunks (i.e., each landmark building being a chunk of visual and spatial information) in their visuospatial memory.

To investigate how the number of landmarks shown on mobile maps can affect cognitive load and spatial learning, we selected three, five, and seven landmarks as a manipulation of low, medium, and high cognitive load conditions, respectively. A prior study by Cheng et al. (2022b) found that *landmark recognition and route direction memory improved when the number of presented landmarks increased from three to five*, while learning performance did not increase further when *seven landmarks* were depicted on the mobile map. Moreover, our prior study assessed cognitive load while participants consulted maps across the three landmarks conditions (3 vs. 5 vs. 7 landmarks), and found that *cognitive load during map consulting increased in the 7-landmark condition* compared to the 3- and 5-landmark conditions.

Previous research has also shown that cognitive load for one attended task may spill over to another subsequent task (Bednar et al., 2012; Liu et al., 2019; Felisberti and Fernandes, 2022). In the study by Felisberti and Fernandes (2022), the cognitive load induced by a series of cognitive tasks was spilled over into the subsequent simulated driving task. In the assisted-navigation context, mobile maps with a good design (e.g., supportive landmark and route information) can assist navigation and spatial learning, and thus may reduce navigators’ cognitive effort when they are navigating and learning the environment. Therefore, the increased cognitive load related to viewing and learning landmarks shown on a mobile map display may also influence cognitive load during navigation through the environment, even if the navigator is no longer attending to the mobile map display.

Moreover, navigation contains both locomotion and wayfinding components (Montello, 2005) and locomotion through the environment occupies most of pedestrians’ time during during navigation and wayfinding (Brügger et al., 2019). It is thus important to disentangle the periods of cognitive load during locomotion through the environment from those periods that relate to cognitive load during map-viewing events.

### 1.4. Assessing cognitive load through brain activity

To assess cognitive load during navigation, we employed electroencephalography (EEG), which records electrical activity originating from the human brain in real time with high temporal resolution by placing electrodes on the head surface. EEG has the advantage of assessing cognitive processing directly through brain activity, compared to other psychophysiological measures (e.g., eye-tracking or electrodermal activity). Moreover, EEG records brain activity in the background without interfering with the primary task. This is unlike behavioral assessments that add another task, as done in dual-task paradigms [where participants complete a cognitive task with different difficulty levels while performing a navigation task, e.g., (Credé et al., 2020)], which would require individuals to respond and consequently interrupt the navigation task.

EEG recordings require event markers that indicate when notable events such as stimulus presentation or participant responses occur. These markers allow the segmentation of EEG data according to these events for event-related analysis. However, visual inputs to participants constantly change when they navigate in a naturalistic environment. In such cases, there is little control over stimulus presentation, and it is thus challenging to add notable event markers based on stimulus presentation. Kalantari et al. (2022) leveraged navigators’ gaze fixation on navigational signs indicating the directions to an ambulatory care unit or to an information desk in a virtual hospital as EEG event markers to study the effect of different interior designs on wayfinding in a hospital facility. Such kinds of event markers (gaze fixation) are meaningful for navigation experiments in environments that contain navigation task-relevant signage and respective feature labeling that navigators are intended to read during navigation. In doing so, the markers will yield long fixation durations (e.g., 1500 ms) compared to incidental glances on unlabeled features. However, this approach might not be easily applicable to outdoor environments that have no explicit labeling and/or navigation-relevant signage such as in open spaces (i.e., residential areas, parks, etc.), where navigators tend to have shorter fixation durations (∼290 ms; Enders et al., 2021). Other methods of event generation, such as adding concurrent tasks to mark participant responses might interrupt participants’ continuous navigation task performance in naturalistic settings and add unwanted affect and arousal interferences. Therefore, a different set of event markers is needed when examining brain activity during navigation in ecologically valid urban environments.

### 1.5. Eye blinks as event markers in naturalistic settings

Previous research has found that spontaneous eye blinks are associated with cognitive load, and especially during the processing of complex visual scenes (Wascher et al., 2014; Valtchanov and Ellard, 2015). When individuals open their eyes after a blink, they receive an influx of visual information, leading to brain activity related to visual processing. Past studies have found that blinks are more likely to occur with higher frequency after a period of blink suppression (e.g., during attentional focus) or when the processing mode changes (e.g., attention re-allocation) (Wascher et al., 2014, 2022). Blinks are thus considered to reflect attentional resource allocation (Stern et al., 1984). Additionally, as eye blinks are generated naturally by users and easily measured with EEG without additional equipment, they could be particularly useful as event markers that indicate cognitive load in naturalistic settings without disrupting continuous task performance (Wascher et al., 2014). Studies investigating blink-related brain activity are thus crucial to validate the use of blinks as event markers to assess cognitive load. However, most research linking eye blinks to cognitive load has focused on characteristics of eye blinks such as the number of blinks and blink deflection while less research has analyzed brain activity related to eye blinks (Orchard and Stern, 1991; Pivik and Dykman, 2004; Valtchanov and Ellard, 2015). Indeed, only a few studies have examined the neuronal processes related to eye blink events (Wascher et al., 2014, 2022; Wunderlich and Gramann, 2020). More research is thus needed that investigates brain activity during eye blinks when individuals perform cognitive tasks to validate the use of eye blinks as indicators of cognitive load and identify the cognitive processes following spontaneous eye blinks.

### 1.6. Blink event-related potentials (bERPs)

Previous research found that event-related potentials (ERPs) occur after eye blinks (Berg and Davies, 1988; Wascher et al., 2014, 2022; Wunderlich and Gramann, 2020). Importantly for the present study, a previous study that used EEG to assess brain activity while using eye blinks as event markers has identified blink event-related potentials (bERPs) associated with the performance of a cognitive task (Wascher et al., 2022). Specifically, the authors compared bERPs during the performance of different tasks (i.e., standing vs. walking on a meadow vs. walking while traversing an obstacle course in a natural environment) while participants were processing auditory information. They found a larger occipital N1, an early negative-going component with a peak latency of about 160 ms in the visual cortex (Näätänen, 1992), during walking compared to standing and traversing an obstacle course, suggesting differences in bottom-up visual perception.

The authors also found a significantly less pronounced amplitude in the fronto-central N2 and parietal P3 with increasing walking demands, indicating that fewer cognitive resources were available for auditory information processing. The blink-related fronto-central N2 (measured at electrodes Fz and FCz) is a negative-going component that occurs around 200 ms after blink maximum (i.e., when the eyes are fully closed) and has been proposed to be associated with cognitive control and an indicator of task demand (Wascher et al., 2014, 2022). The blink-related posterior P3 (measured at electrodes Pz, POz, Oz) is a positive-going component that occurs around 250 ms after blink maximum, and is an indicator of cognitive resource allocation (Wascher et al., 2014, 2022). This is similar to the stimulus-evoked posterior P3, which has been shown to be a reliable indicator of resource allocation during cognitive processing and a valid index of cognitive load (Kok, 2001; Scharinger et al., 2017). Specifically, increased cognitive load requires more resources for cognitive processing, leading to an increased P3 amplitude. Increasing levels of cognitive load (i.e., low to medium to high) may thus lead to increases in blink-related P3 amplitude in the parieto-occipital regions.

### 1.7. The present study

In a previous conference short paper, Cheng et al. (2022a) assessed blink-related brain potentials across the three landmarks conditions over the *entire map-assisted navigation task, including map reading*. Because Cheng et al. (2022a) did not separate eye blink events during the locomotion portion of the navigation task from the map-viewing events during the navigation task, it is not yet clear whether depicting different numbers of landmarks on mobile maps led to changes in cognitive load during goal-directed navigation or vice versa. Additionally, in the present study, we were interested in separating the locomotion phase in the environment from the map-viewing events to better disentangle the potential overlap between map-onset brain potentials and blink-related brain potentials.

In the present study, similar to the prior conference contribution with preliminary results by Cheng et al. (2022a), we investigated blink-related brain potentials to assess how the number of landmarks displayed individually at specific intersections on a mobile map would affect navigators’ cognitive load during navigation. In this study, however, we assessed blink-related potentials during the locomotion portion of the navigation task *separately* from the map viewing periods. We also analyzed blink-related frequency changes to investigate cognitive load indicated by the frequency domain of the EEG data to assess their convergence with bERPs. Finally, to increase statistical power of the within-subject analysis, the present study used linear-mixed effect models to examine the identified differences in brain activity between the landmark conditions.

We utilized a within-participant design with three different numbers of landmarks (3 vs. 5 vs. 7). We selected visually salient buildings at intersections along a route as landmarks. Participants were asked to navigate to predetermined destinations in three different virtual environments with the assistance of a mobile map that provided turn-by-turn directions. Participants were also instructed to remember landmarks from a first-person view that were either seen in the traversed virtual urban environment or on the mobile map during navigation. After each navigation trial in each city, participants’ spatial knowledge of the traversed environment was assessed.

We hypothesized no difference in the occipital blink-related N1 amplitude between the landmark conditions, as the neural processes underlying bottom-up visual perception in the identical environments were not expected to change. This is because the assessed landmarks differed on only the mobile map displays and not in the traversed environments. We also hypothesized that displaying more landmarks on a mobile map would increase cognitive load during navigation, as indicated by bERPs—a more pronounced N2 amplitude in the fronto-central region, and a more pronounced P3 amplitude in the parieto-occipital region. Because little work has investigated blink-related frequency changes with respect to changes in cognitive load (Wascher et al., 2016, 2022), we also explored potential differences in frontal theta power changes and parietal alpha power changes across the three landmark conditions. We hypothesized that fronto-central theta power would increase and parieto-occipital alpha power would decrease with increasing numbers of displayed landmarks (Wascher et al., 2016, 2022) due to increased cognitive load.

## 2. Materials and methods

### 2.1. Participants

Forty-nine participants (29 females) with ages ranging from 18 to 35 years (*M* = 25.6 years, *SD* = 4.09) took part in the study. Exclusion criteria consisted of having a history of a neurological or mental disorder. One participant was excluded due to self-reported mental illness during the experiment and requested to have their data excluded. All participants were compensated with 30 CHF for their participation. All participants gave informed consent in compliance with the ethical standards of the University of Zurich Ethics Board, the Swiss Psychological Society, and the American Psychological Association.

The analyzed data in the current study were collected from the same participants as reported in Cheng et al. (2022a,b). For all three studies, we excluded the data of one participant because of the presence of severe artifacts in their EEG data.

### 2.2. Experimental design

**FIGURE 1:**
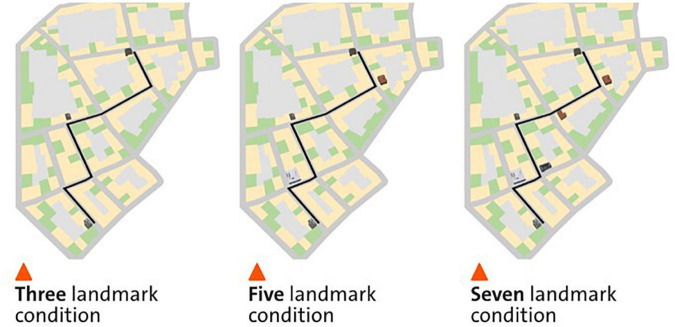
The three different landmark conditions in a virtual city. The **left**, **middle**, and **right** panels depict the map condition with three, five, and seven landmarks displayed on the mobile map, respectively. The figure is adapted from Cheng et al. (2022b).

We adopted a within-participant design with three conditions, showing either three, five, or seven landmarks on the mobile map while participants navigated a predefined navigation route (Figure 1). The three conditions were counterbalanced across three different virtual cities. In the 3-landmark condition, a building at the start location, at the destination, and a building at the third intersection were displayed on the mobile map (see Figure 1A). In the 5-landmark condition, the two additional buildings at the first and fourth intersection were visualized on the map respectively, compared to the 3-landmark condition (see Figure 1B). In the 7-landmark condition, the two additional buildings at the second and fifth intersections were displayed on the map respectively, compared to the 5-landmark condition (see Figure 1C). The building positions for each landmark condition were selected to ensure that the landmarks were evenly spaced along the route.

### 2.3. Experimental task

**FIGURE 2:**
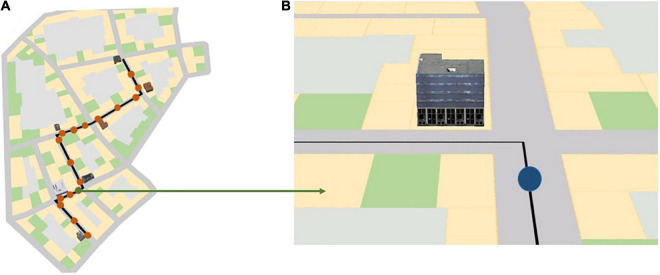
**(A)** Red dots along the black navigation route indicate the 17 map pop-up spots during navigation in the three different landmark conditions; **(B)** a mobile map that rotates along with the participant’s head direction, as seen by the participant at the location of the green dot in panel **(A)**. The blue dot in panel **(B)** indicates the participant’s current location in the virtual city. The black line indicates the path the participant needs to follow. Depending on the landmark condition, three, five, or seven 3D landmarks are shown on the map at a turning intersection. The figure is adapted from Cheng et al. (2022b).

The navigation portion of the experiment consisted of three blocks and a 2-min break between these blocks. All participants completed all three blocks one after the other. Each block consisted of a map-assisted navigation task in a virtual urban city and spatial learning tests immediately after navigation in each city. During the navigation phase, participants were instructed to follow the route indicated on the mobile map as quickly as possible to a specific destination and to learn the landmarks at the intersections along the route that were displayed on the map (Figure 2). Participants were also told that some of the landmarks at the intersections that were not visualized on the mobile map would also be tested after navigation. After navigating through each city, participants’ spatial knowledge was tested. To assess participants’ different levels of spatial knowledge acquisition (i.e., landmark knowledge, route knowledge, and survey knowledge; Siegel and White, 1975; Chrastil, 2013), we employed a landmark recognition test, a route memory test, and a judgment of relative direction (JRD) test at the end of each navigation task in each city.

**FIGURE 3:**
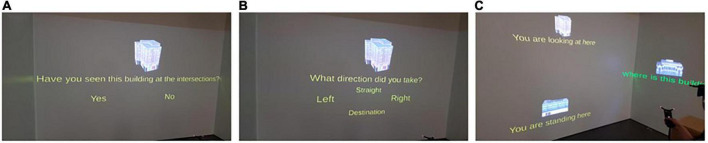
The three panels depict how participants responded to the spatial learning tests in the CAVE using a 3D pointing device after navigation: **(A)** The landmark recognition test, **(B)** the route direction test, and **(C)** the JRD test, respectively. The figure is adapted from Cheng et al. (2022b).

The *landmark recognition test* assessed participants’ ability to discriminate between landmarks seen at intersections (including the starting building and the destination) along the route compared to novel buildings that were not seen along the route (Huang et al., 2012; Stites et al., 2020; Wunderlich and Gramann, 2020, 2021; Kim and Bock, 2021). Participants were asked whether they had seen the shown landmarks along the route and responded with either “yes” or “no” (Figure 3A).

The *route direction test* assessed participants’ direction memory in reference to the assessed landmarks seen at intersections to prevent participants from simply guessing (Huang et al., 2012; Wunderlich and Gramann, 2020, 2021; Kim and Bock, 2021). Hence, for landmarks that participants answered “yes” in the prior landmark recognition test, they were subsequently asked to indicate the route direction they took at the intersection with the associated landmark. Response options included the forced choice of either “left,” “right,” “straight,” or “destination” (Figure 3B).

The *JRD* test assessed participants’ knowledge of the relative spatial (e.g., angular) directions of three given landmark locations (Zhang et al., 2014; Huffman and Ekstrom, 2018). Participants were asked to imagine standing at a first landmark while facing a second landmark and to point to a third landmark (Figure 3C).

Further details about the procedure, including a training trial in the virtual environments, and the spatial learning tests, can be found in Cheng et al. (2022b).

While participants were performing the navigation task, their brain activity was measured using a 64-channel EEG device with active electrodes (LiveAmp, Brain Products GmbH, Gilching, Germany). EEG was recorded at a 500 Hz sampling rate with input impedance set at below 10 kOhm.

### 2.4. Experimental stimuli and apparatus

**FIGURE 4:**
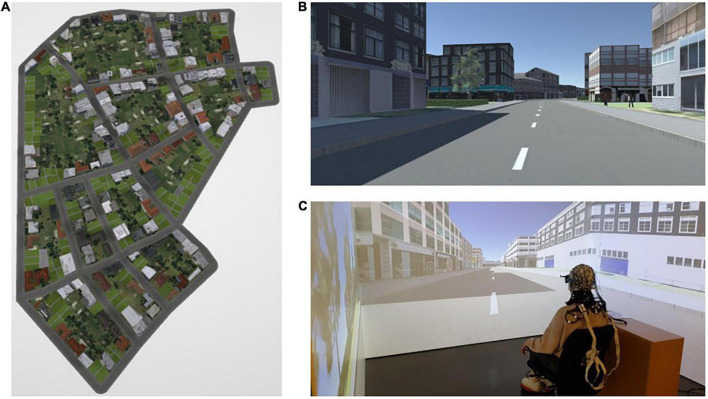
**(A)** Bird’s eye view of one of the virtual cities; **(B)** participants’ view of the environment during navigation; and **(C)** a participant seated on a chair approx. 30 cm away from the center of the VR system (CAVE), placed her feet on a foot-operated controller, and was equipped with an EEG device during the navigation experiment. The figure is adapted from Cheng et al. (2022b).

**FIGURE 5:**
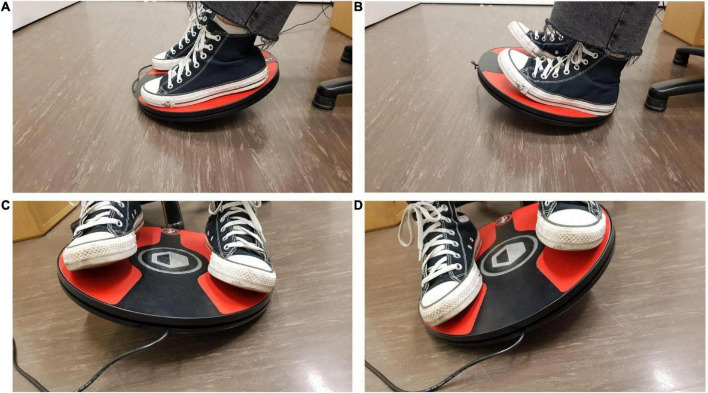
The participant is titling the foot controller with their feet to **(A)** move forward and **(B)** backward through the virtual environment, respectively. The participant is titling the foot controller with their feet to **(C)** the left and **(D)** to the right to turn their heading direction in the urban virtual environment toward the left and right, respectively.

Three virtual cities were designed in ArcGIS City Engine 2018.0 and displayed on a three-sided, stereo cave automatic virtual environment (CAVE) using Unity 2018.4 LTS (Figures 4A, B). Participants moved by using a foot-operated controller (Figure 4C) through the virtual environment displayed in the CAVE. Tilting the foot controller toward the front and back resulted in forward and backward movement in the urban environment, respectively (Figures 5A, B). When tilting the foot controller toward the left and the right, participants could turn to the left and to the right, respectively (Figures 5C, D).

Each city contained a predefined route to be followed. The current part of the route was shown on a mobile map projected on the center screen of the CAVE during navigation. This map provided navigators’ current location and turn-by-turn instructions by displaying the route as a black line and rotated along with the navigators’ heading direction. The map appeared 17 times and each instance lasted for 5 s; during this time the city faded away and participants’ movement was disabled (Figure 2A). The map appeared shortly before the participants arrived at the intersections, after they passed the intersections, and in the middle of the straight segments where they first saw the next intersection. This simulated navigators’ mobile map use during wayfinding in the real world. While landmarks were always visible in the virtual environment, depending on the landmark condition, the chosen landmark at the intersection was shown in 3D on the mobile map, as seen in the environment (Figure 3B).

### 2.5. EEG data preprocessing

We used the BeMoBIL pipeline (Klug et al., 2022) to preprocess the raw EEG data in the MATLAB toolbox EEGLAB (Delorme and Makeig, 2004). This pipeline is designed to automatically preprocess EEG data, optimized for later independent component analysis (ICA, Makeig et al., 1995). It also supports the improvement of the signal-to-noise ratio, which is especially critical in EEG datasets that are collected while participants are moving. We first removed the non-experimental segments from the raw EEG datasets, before submitting the raw data into the BeMoBIL pipeline. We first downsampled the raw EEG data to 250 Hz. Then, we applied the *ZapLine Plus* function to remove spectral peaks at 50 Hz, corresponding to the power line frequency (Klug and Kloosterman, 2022). We identified noisy channels using the automated rejection function *clean_artifacts* from EEGLAB with ten iterations. We removed the bad channels that were detected more than four times out of the ten iterations and interpolated them using a spherical spline function. We then re-referenced the data to the averaged reference across the whole set of electrodes. On this cleaned dataset, we conducted ICA using an adaptive mixture independent component analysis (AMICA) algorithm (Palmer et al., 2011) with the recommended parameter values from Klug and Gramann (2021). The AMICA decomposition uses a log-likelihood removal of samples that are not corresponding to the algorithm’s estimate of the model fit. We applied five iterations in AMICA cleaning with three standard deviations as removal criterion. Besides this AMICA-inherent time-domain cleaning, high-pass filtering with a 2-Hz cutoff and automatic time-domain rejections were performed before AMICA computation, to improve the ICA decomposition. For each resultant independent component (IC), we computed an equivalent current dipole (ECD) model using DIPFIT routines from EEGLAB (Oostenveld and Oostendorp, 2002). This computed information including rejections and dipole fitting resulting from AMICA is copied back to the preprocessed but unfiltered EEG dataset with the BeMoBIL pipeline, considering that final EEG measures (e.g., ERPs) may require a lower cutoff-filtering on the EEG data (Klug and Gramann, 2021; Klug et al., 2022).

We applied a 0.5–30 Hz pass filter to suppress slow drifts and high-frequency activity in the EEG signal. We then removed the EEG recordings during the map presentation events (always a 5 s time window), that is, when participants were shown the mobile map, the virtual urban environment faded away, and their movements through the environment were disabled.

### 2.6. Eye blink detection

To detect and extract brain activity related to eye blinks, we followed the protocol established by Wunderlich and Gramann (2020). First, the component representing vertical eye movements was identified and filtered using a moving median with a window size of 20 sample points (80 ms). Then, blinks were identified in the vertical eye movement component using Matlab’s *findpeaks* function [min. peak width = 5 time points (20 ms); max. peak width = 65 time points (260 ms); min. peak height = peak heights ≥ 96 percentile; min. peak prominence = peak prominence ≥ 97 percentile; min. peak distance = 25 time points (100 ms)]. Event markers were placed at time points of maximum blink deflections. We then used the ICLabel algorithm (Pion-Tonachini et al., 2019) with the default classifier to classify the resultant ICs in classes representing, e.g., eye, brain, or other components. Based on this classification, we removed ICs from the data that were classified as unlikely to represent brain activity (i.e., probability below 30%), following the approach suggested by Wunderlich and Gramann (2020) for Mobile Brain/Body Imaging (MoBI) EEG data, as ICLabel was mainly trained on stationary datasets with only few mobile EEG or MOBI datasets for training the IC classifiers. As such, movement-related activity stemming from the neck musculature and other such sources are usually not well classified. Moreover, increasing the number of movement-related brain and non-brain sources, while having only a limited number of channels and thus only limited degrees of freedom for the decomposition, can increase the likelihood of brain sources being mixed with other sources. This in turn can result in non-standard IC topographies and spectra. We therefore chose a conservative threshold of 30% to avoid excluding any potentially useful brain sources.

### 2.7. bERP extraction

To extract bERPs, we used the Unfold toolbox (Ehinger and Dimigen, 2019) on blink events during the navigation phase only (i.e., not during map reading). The unfolding technique allows for a regression-based separation of overlaying event-based brain activity. As blink rate is high in this naturalistic navigation setting in the open-world virtual environments (Enders et al., 2021), this toolbox would be useful for separating overlapping blink-related brain activity (i.e., two blinks happening very close to each other) in our study.

We first created a design matrix with blink events and 65 channels. Information on the different landmark conditions (3, 5, and 7 landmarks) was entered into the regression formula *y = 1 + cat(landmark)*. We also applied continuous artifact detection and rejection with an amplitude threshold set at ± 80 microVolts (μVs) during unfolding, to reject the segments with noisy artifacts from our continuous EEG datasets. The design matrix was then time-expanded according to the time limits of -500 to 2000 ms with respect to blink events. A general linear model was then fitted to solve for the intercept and beta values with a baseline correction at -500 to -200 ms preceding the blink event (Wascher et al., 2014; Wunderlich and Gramann, 2020).

**FIGURE 6:**
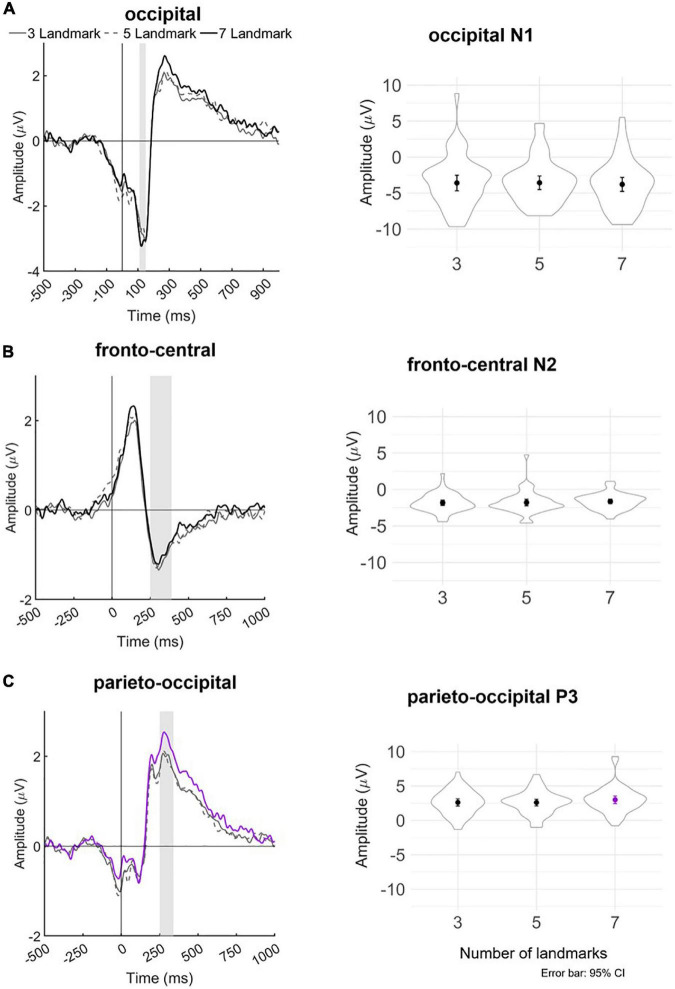
Left panel: Grand averaged amplitudes of blink-related ERPs for each experimental landmark condition at the **(A)** occipital lead (Oz), **(B)** fronto-central leads (Fz and FCz), and **(C)** parieto-occipital leads (Pz, POz, and Oz). The blink-related ERP signals served as the basis for individual peak detection—vertical bars shaded in gray indicate the time windows in which the respective minima or maxima of the bERPs were identified. Right panel: Violin plots depicting the distribution of detected peak amplitudes together with mean and ±1.96 standard error (i.e., 95% CI) in each landmark condition for occipital N1, fronto-central N2, and parieto-occipital P3. Line and mean plotted in purple in the bottom panel indicate statistical significance at *p* < 0.05.

We then recovered the modeled bERPs from the unfolded intercept and beta values using matrix multiplication (Ehinger and Dimigen, 2019) for the electrodes of interest (Fz, FCz, Pz, POz, and Oz; Wascher et al., 2014, 2016) for statistical analysis using individual peak detection. Based on visual inspection of the grand averaged bERP plots (Figure 6: left panel), we selected the following time windows for individual peak detection with the neighboring +3 and -3 sample points around the detected peaks (i.e., seven data samples in total; Takeda et al., 2014; Wunderlich and Gramann, 2020; Sugimoto F. et al., 2022): the N1 amplitude was extracted 110–150 ms after blink maximum and averaged across occipital (Oz). The N2 amplitude was extracted 250–390 ms after blink maximum and averaged across fronto-central leads (Fz and FCz). The P3 was extracted 250–340 ms after blink maximum and averaged across parieto-occipital leads (Pz, POz, and Oz).

### 2.8. Frequency-domain processing

We additionally conducted frequency-domain analyses for exploratory purposes. After removing independent components unlikely to represent brain activity following blink detection, we also extracted segments of -500 to 2000 ms with respect to blink events. We replicated the approach by Cheng et al. (2022b) to calculate fronto-central (FC1, FCz, FC2) theta (4–7.9 Hz) ERS and parieto-occipital (PO3, POz, and PO4) alpha (8–12.9 Hz) ERD during navigation. To obtain baseline power, we calculated power indices during the time before the navigation experiment started, that is, when participants were sitting on a chair and viewing a dark blue screen of the front CAVE wall in front of them. The baseline started when participants put on the 3D stereo glasses and ended when the urban environment was fully loaded and visible on all walls of the CAVE. The baseline phase varied from 6 to 20 s, including a period when participants felt ready to start the navigation portion of the experiment. We extracted the baseline epochs with a length of 1 s from this pre-navigation experiment phase. Baseline epochs had 200 ms overlap with subsequent epochs. We obtained ERS (positive values) and ERD (negative values) by using the following formula (Pfurtscheller and da Silva, 1999):


E⁢R⁢S⁢o⁢r⁢E⁢R⁢D=⁢(r⁢e⁢l⁢a⁢t⁢i⁢v⁢e⁢p⁢o⁢w⁢e⁢r⁢d⁢u⁢r⁢i⁢n⁢g⁢n⁢a⁢v⁢i⁢g⁢a⁢t⁢i⁢o⁢n-r⁢e⁢l⁢a⁢t⁢i⁢v⁢e⁢p⁢o⁢w⁢e⁢r⁢d⁢u⁢r⁢i⁢n⁢g⁢b⁢a⁢s⁢e⁢l⁢i⁢n⁢e)/r⁢e⁢l⁢a⁢t⁢i⁢v⁢e⁢p⁢o⁢w⁢e⁢r⁢d⁢u⁢r⁢i⁢n⁢g⁢b⁢a⁢s⁢e⁢l⁢i⁢n⁢e.

### 2.9. Statistical analyses: Multilevel linear regression

To assess the effect of the landmark conditions (3 vs. 5 vs. 7 landmarks) on cognitive load during navigation, we entered the peak amplitudes of N1, N2, and P3 in R version 4.0 (Bates et al., 2011) and ran for each bERP a linear regression model, with the α level set at 0.05 for all analyses. Multilevel modeling is a generalization of regression analysis and is able to separately estimate the effects of an individual predictor and its group-level mean (Gelman, 2006) while ignoring missing values in predictors (Fitzmaurice and Molenberghs, 2008). This allows us to perform a within-participant analysis and include two participants with incomplete data in the analysis.

We adopted the mixed-effects regression as a hypothesis-driven confirmatory approach and modeled the effect of the number of landmarks on cognitive load indicated by EEG measures (i.e., bERPs: peak amplitudes of N1, N2, and P3; frequency band power: theta ERS and alpha ERD) separately. We built and performed the multilevel models using the *lmer4* package in R version 4.0 (Bates et al., 2011). Following the recommendations by Barr et al. (2013) on multilevel models for confirmatory hypothesis testing, we first identified the maximal random effects structure by including by-participant intercepts and slopes in the random structure, based on our within-participant experimental design. Next, we simplified the maximal random-effects model by first excluding random slopes and then random intercepts until the model converged. The first model that converged included by-participant intercepts in the random-effects structure. The following equation described our multilevel model:


$$P3amplitude_{pi}\text{ = }\beta_{\text{0}}\text{ + }\beta_{1}^{*}\;Condition_{i}\text{ + }P_{\text{0}}{}_{p}\text{ + }e_{pi}$$

where *P3 amplitude_*pi*_*, for participant *p* and item *i*, is related to a reference level *via* fixed-effect β_0_ (the intercept), a landmark condition effect *via* fixed-effect β_1_ (the slope), the deviation from β_0_ for participant *p*, and the observation-level error *e_*pi*_.* In this model, parameters β_0_ and β_1_ represent fixed effects, and the parameter *P*_0p_ represents random effects.

## 3. Results

### 3.1. Behavioral results

#### Number of blinks

We normalized the number of blinks by condition for each participant by dividing the number of blinks for that condition by the mean number of blinks made by that participant across conditions, to reduce inter-subject variability. The normalized number of blinks is lowest in the 5-landmark condition [5 vs. 3: *beta* = -0.10, 95% CI (-0.19, -0.01), *p* = 0.023; 5 vs. 7: *beta* = -0.12, 95% CI (-0.21, -0.03), *p* = 0.008]. There is no significant difference in the number of blinks between the 3- and 7-landmark conditions [7 vs. 3: *beta* = 0.02, 95% CI (-0.07, 0.10), *p* = 0.69]. No significant difference between the three landmark conditions on the absolute number of blinks is observed (*p*s > 0.124). Table 1 presents the means and standard errors of the normalized and absolute number of blinks in the three landmark conditions.

**TABLE 1:** Means and standard errors of the normalized and absolute number of blinks for each landmark condition.

Number of blinks	LM condition	Mean	Std. error
Normalized	3	1.03	0.032
	5	0.93	0.028
	7	1.05	0.033
Absolute	3	106.0	13.0
	5	97.8	10.8
	7	111.0	10.0

#### Navigation time

Participants navigated from the starting position to the destination in the three cities on average for 8.11 min (*SD* = 1.63 min). No significant difference in navigation time is observed between the three landmark conditions (*p*s > 0.507).

### 3.2. bERP results

No significant difference is found in N1 amplitude in the occipital region between the three landmark conditions (*p*s > 0.256). No significant difference is observed in the N2 amplitude in the fronto-central region between the landmark conditions (*p*s > 0.395).

The linear mixed-effect models reveal that P3 amplitude in the parieto-occipital region in the 7-landmark condition is significantly greater than in the 3- and 5-landmark conditions. P3 amplitude increases by 40% on average from the 3-landmark to 7-landmark condition [7 vs. 3: β = 0.40, 95% CI (0.01, 0.79), *p* = 0.046] and by 41% on average from the 5-landmark to 7-landmark condition [7 vs. 5: β = 0.41, 95% CI (0.02, 0.80), *p* = 0.040]. Contrary to our hypothesis, there is no significant difference between the 3- and 5-landmark conditions [5 vs. 3: β = -0.01, 95% CI (-0.40, 0.38), *p* = 0.959].

Figure 6 depicts the mean bERP amplitudes and the detected peak amplitude for each landmark condition. Table 2 provides a more comprehensive overview of the multilevel model coefficients. Table 3 provides a comprehensive overview of the means and standard errors of the bERPs and the detected peak amplitude for each landmark condition.

**TABLE 2:** Regression coefficients of peak amplitudes of the N1 at the occipital lead (Oz), N2 at fronto-central leads (Fz and FCz), and P3 at parieto-occipital leads (Pz, POz, and Oz) across pairwise contrasts of the landmark conditions.

ROIs	Peak type	Time window (ms)	LM condition contrast	β	95% CI	*p*
Occipital	N1	110–150	5 vs. 3	-0.16	–0.82–0.51	0.649
			7 vs. 5	-0.23	–0.90–0.43	0.494
			7 vs. 3	-0.39	–1.06–0.28	0.256
Fronto-central	N2	250–390	5 vs. 3	0.01	-0.35–0.37	0.969
			7 vs. 5	0.15	–0.21–0.51	0.415
			7 vs. 3	0.16	–0.20–0.52	0.395
Parieto-occipital	P3	250–340	5 vs. 3	-0.01	–0.40–0.38	0.959
			7 vs. 5	0.41	0.02–0.80	**0.040**
			7 vs. 3	0.40	0.01–0.79	**0.046**

*P*-values in bold indicate significant differences at *p* < 0.05.

**TABLE 3:** Means and standard errors of peak amplitudes of the N1 at the occipital lead (Oz), N2 at fronto-central leads (Fz and FCz), and P3 at parieto-occipital leads (Pz, POz, and Oz) for each landmark condition.

ROIs	Peak type	Time window (ms)	LM condition	Mean	Std. error
Occipital	N1	110–150	3	-3.57	0.56
			5	-3.56	0.48
			7	-3.80	0.50
Fronto-central	N2	250–390	3	-1.82	0.18
			5	-1.78	0.23
			7	-1.64	0.16
Parieto-occipital	P3	250–340	3	2.61	0.26
			5	2.60	0.25
			7	3.00	0.28

### 3.3. Exploratory analyses

#### Theta ERS/Alpha ERD

No difference in frontal theta ERS and parietal alpha ERD is observed between the landmark conditions (*p*s > 0.144).

#### Correlation analysis

We additionally performed an exploratory correlation analysis (Pearsons’ correlations coefficients, two-tailed) between the P3 amplitude and spatial learning performance (i.e., landmark recognition, route direction memory, and JRD response errors) and found no significant correlations between the P3 amplitude and spatial learning performance (*p*s > 0.2).

## 4. Discussion

The present study examined cognitive load measured by EEG during map-assisted navigation in virtual environments while depicting either 3, 5, or 7 landmarks out of 7 chosen landmarks from the environment on the mobile maps. Changes in cognitive load during navigation were assessed with blink-related brain potentials in the fronto-central and parieto-occipital regions. We found that P3 amplitude was significantly higher in the 7-landmark condition compared to the 3- and 5-landmark conditions.

### 4.1. bERP characteristics during navigation

Our blink-related potential at fronto-central leads presented a positive component (P1) and a subsequent negative component (N2). The blink-related ERP at parieto-occipital leads presented first a negative component (N1), followed by a positive peak (P2) and a negative component (N2), and finally a P3-like component. Lastly, the blink-related potential at the occipital lead presented a clear N1 component followed by a P3-like component. The P2 component at the occipital lead was not clearly presented. The general characteristics of our blink-related N1, N2, and P3 generally are in line with stimulus-evoked N1, N2, and P3 in previous ERP research (Luck, 2012), as well as those reported in previous studies that examined bERPs (Wascher et al., 2014, 2022; Wunderlich and Gramann, 2020). This suggests that using blinks to parse brain activity might be a valid method to assess cognitive load in an ecological setting.

### 4.2. bERPs—Cognitive processing

#### N1—Bottom up visual processing

The lack of significant difference in occipital N1 amplitude between the landmark conditions suggests that visualizing different numbers of landmarks on the mobile map does not influence navigators’ bottom-up visual perception when they move through the virtual environments. The variances of the detected peaks in the N1 component are larger compared to those in the N2 and P3 components. This is also in line with the relatively larger variance of the occipital N1 component in previous studies (Wascher et al., 2014, 2022).

#### N2 and P3—Cognitive load

We did not observe any difference between the experimental conditions on the blink-related fronto-central N2 amplitude, which is associated with top-down processing (Wascher et al., 2014, 2022). This might be because the fronto-central N2 component is sensitive enough to distinguish cognitive load and no load conditions (Wascher et al., 2014, 2022) but not sensitive enough to distinguish between different levels of cognitive load. Another interpretation might be because the stimulus-evoked N2 is usually associated with cognitive control and mismatch (for a review, Folstein and Van Petten, 2008), which might not be relevant to our current experimental design. Future research should further examine the relationship between blink-related N2 and cognitive load.

Previous literature on stimulus-evoked ERPs has established a positive relationship between parieto-occipital P3 amplitude and cognitive effort exertion (for a review: Kok, 2001). Similarly, the blink-related posterior P3 component is proposed to reflect attentional resource management in a recent study by Wascher et al. (2022), whereby a decreased blink-related P3 amplitude indicates fewer attentional resources being used on the task. Our finding indicates that more attentional resources are expended when navigating through the environment in the 7-landmark condition, compared to the 3- and 5-landmark conditions. However, participants’ spatial learning performance does not further improve from seeing seven landmarks on the mobile map. These findings together suggest that participants’ attentional resources might not be effectively directed to relevant stimuli in the environment in the 7-landmark condition, because the 7 landmarks depicted on mobile maps lead to cognitive overload during map reading. To examine this interpretation, future work should employ an eye-tracker to analyze navigators’ fixations on relevant or irrelevant stimuli in the environment (see section “Limitations and future work” for a more detailed discussion).

Moreover, the current finding related to P3 amplitude is also consistent with the finding of a related paper (Cheng et al., 2022b), which analyzed cognitive load while participants viewed the mobile map (i.e., not while they were moving through the environment). Parieto-occipital P3 amplitude during map viewing was also more pronounced in the 7-landmark condition compared to the 3- and 5-landmark conditions (Cheng et al., 2022b). Taken together, the results suggest that cognitive load during map reading might have spilled over into navigation or vice versa, as evidenced by greater P3 amplitude during both navigation and map reading when seven landmarks are visualized on the mobile map. This is consistent with previous studies showing that cognitive load in one task can affect cognitive load in another task (Bednar et al., 2012; Felisberti and Fernandes, 2022). This pattern of increased P3 amplitude in the 7-landmark condition is also consistent with blink-related P3 amplitude during the entire wayfinding phase, which comprised both navigation and map-consultation (Cheng et al., 2022b). Based on our findings, it seems that displaying five landmarks one by one along a route provides the best design for mobile maps. In doing so, it improves spatial learning without taxing additional cognitive resources during map reading and goal-directed locomotion through the virtual environment.

### 4.3. Blink behavior

In our study, we found the lowest numbers of blinks in the 5-landmark condition, compared to the 3- and 7-landmark conditions. Previous literature suggests that blink bursts are associated with high cognitive load (Siegle et al., 2008), and possibly reflect more cognitive resources being used in stimulus-related cognition (Ohira et al., 1998). Valtchanov and Ellard (2015) also found that when participants were viewing environmental scenes, fewer blinks were associated with lower cognitive load. Our findings on the normalized number of blinks suggest that participants might have the lowest cognitive load while navigating in the environment when five landmarks are depicted on the map. However, this pattern is different from the pattern shown in parieto-occipital P3 amplitude, as discussed in the above section. To further investigate and interpret the relationship between blink behavior, such as the number of blinks, and cognitive load, future studies should also include other blink-related measures collected with eye tracking and/or pupillometer, to detect blinks more accurately and assess other blink-based measures (e.g., blink duration, blink intervals) more deeply.

### 4.4. Contributions to navigation system development

The contributions of our current research to the field of navigation system development are twofold. First, our current study makes a methodological contribution to the field of human-computer interaction (HCI), part of which investigates users’ interactions with navigation systems (e.g., Savino et al., 2020, 2021). In this field, user behavior and eye-tracking systems are commonly employed to examine how users interact with navigation devices (Göbel et al., 2019). Neuroscientific methods can be used to complement existing methods used in HCI to obtain an in-depth insight into cognitive states and cognitive processing during navigation. Furthermore, the method of using blinks to parse brain activity makes it possible to directly assess users’ cognitive states without interfering with their primary task (i.e., navigation). Our current study thus provides evidence in the HCI field that blink-related brain activity can be a useful method to investigate users’ cognitive states when they are interacting with mobile applications.

Second, our current research also extends the literature on assisted navigation by showing that depicting different numbers of landmarks on mobile maps influences users’ spatial learning, cognitive load during device use, and during navigation. In recent years, there is increasing attention on employing neurocognitive methods to investigate map-assisted navigation (Cheng et al., 2022b; Liu et al., 2022), although research thus far remains sparse. Among these very few studies, users’ cognitive states were assessed only during map reading and not while navigating through an environment. Our current study suggests a cognitive load spillover effect—cognitive states during map use during navigation outside of map reading might influence each other. Examining both phases helps us to better understand the factors that contribute to cognitive load during map-assisted navigation as a whole and their impact on spatial learning. Our results indicate that mobile map designers and navigation system developers should consider how the processing of presented map information could influence users’ cognitive load during navigation and in turn affect spatial learning in the designs of their mobile navigation applications.

### 4.5. Limitations and future work

The current study provides first evidence of a relationship between the number of landmarks shown on a mobile map and blink-related cognitive load during mobile map-assisted navigation. A worthwhile follow-up question that arises from our findings is whether this relationship is monotonic or discrete. At this stage of the research, we do not know yet whether navigators’ cognitive load increases further when the mobile map displays six landmarks and then plateaus at the seventh landmark, or whether cognitive load continuously increases with more than five shown landmarks. Future studies could follow our paradigm and investigate mobile map displays with six or more than seven landmarks to answer this research question. This will allow a more comprehensive understanding of the relationship between the number of landmarks visualized on a mobile map and cognitive load of navigators and enable the development of a neuroadaptive mobile map that gradually adapts the number of landmarks based on navigators’ cognitive load.

Furthermore, our findings in the current study provide a starting point to examine cognitive load changes during map-aided navigation in virtual environments by analyzing blink-related brain potentials. More future work on map-assisted navigation in the real world with higher ecological validity is needed to apply our findings to the real world. Indeed, although previous studies (Armougum et al., 2019; Kalantari et al., 2021) found that cognitive load level measured by electrodermal activity and self-reported questionnaires during navigation in virtual reality is fairly similar to cognitive load level in the real world, body-based cues (e.g., vestibular and proprioceptive information) in real-world navigation could influence wayfinding and spatial learning (Gramann et al., 2021). In addition, environmental factors (e.g., wind) may influence blink rate. Therefore, future research should consider such factors when designing real-world navigation studies with mobile EEG.

Future research should also combine eye-tracking and EEG to further examine the reliability and validity of blink-related potentials as an assessment of cognitive load during navigation. Eye-trackers provide more information on users’ ocular activity, such as whether they fixate on stimuli in the environment or the navigation device (Kapaj et al., 2021). Such information can help researchers to categorize blinks according to the focal stimuli and contribute to the interpretation of the results of blink-based brain activity.

## 5. Conclusion

The present empirical research on blink-related brain potentials reveals that visualizing landmarks on mobile maps influences navigators’ cognitive load during navigation in virtual environments. Our findings synthesize the fields of cognitive neuroscience, navigation information system design, and brain-computer interface. Combined with findings of map-related cognitive load and spatial learning, our findings suggest that a mobile map with a medium number of landmarks (i.e., five landmarks) seems to be optimal to support spatial learning without overtaxing navigators’ attentional resources during navigation and map reading. Our findings also suggest a cognitive load spillover effect during map-assisted navigation and wayfinding whereby cognitive load during map viewing might have affected cognitive load during navigation in the environment or vice versa. By examining the effect of different numbers of landmarks visualized on mobile maps on blink-related brain activity, the current study demonstrates that blink-related potential analysis is a valid method to assess cognitive load during navigation.

## Data availability statement

The raw data supporting the conclusions of this article will be made available by the authors, without undue reservation.

## Ethics statement

The studies involving human participants were reviewed and approved by the University of Zurich Ethics Board. The patients/participants provided their written informed consent to participate in this study.

## Author contributions

BC, KG, and SF designed the study. BC performed data collection and drafted the manuscript. BC and EL performed data analysis. AW and KG assisted with data analysis. All authors were involved in revising the manuscript and read and approved the final manuscript.
